# Relating X-ray photoelectron spectroscopy data to chemical bonding in MXenes†

**DOI:** 10.1039/d0na01033b

**Published:** 2021-03-01

**Authors:** Néstor García-Romeral, Masoomeh Keyhanian, Ángel Morales-García, Francesc Illas

**Affiliations:** Departament de Ciència de Materials i Química Física, Institut de Química Teòrica i Computacional (IQTCUB), Universitat de Barcelona c/Martí i Franquès 1-11 08028 Barcelona Spain francesc.illas@ub.edu; Department of Physical Chemistry, Faculty of Chemistry, University of Mazandaran Babolsar 47416-95447 Iran

## Abstract

The relationship between core level binding energy shifts (ΔCLBEs), that can be experimentally determined by X-ray photoelectron spectroscopy, and chemical bonding is analyzed for a series of MXenes, a new family of two-dimensional materials with a broad number of applications in nanotechnology. Based on first-principles calculations, the atomic and electronic structure of bare and O-terminated carbide MXene with M_2_C and M_2_CO_2_ (M = Ti, Zr, Hf, V, Nb, Ta, Cr, Mo, and W) stoichiometries are investigated with a focus on trends in the C(1s) and O(1s) ΔCLBEs, including initial and final state effects, along with the series. A rather good linear correlation between the available experimental and calculated C(1s) and O(1s) ΔCLBEs exists, with quantitative agreement when final state effects are included, that validates the conclusions from the present computational approach. The present study shows that ΔCLBEs of bare MXenes are governed by the initial state effects and directly correlate with the net charge on the C atoms. However, for the case of O-terminated MXenes, C(1s) and O(1s) ΔCLBEs exhibit a much less significant correlation with the net charge of either C or O atoms which is attributed to the structural changes induced on the M_2_C moiety by the presence of the O layers and the different stacking sequence observed depending on the MXene composition. The present study shows how and when XPS can be used to extract information regarding the nature of the chemical bond in bare or functionalized MXenes.

## Abstract 2

The relationship between core level binding energy shifts, available through X-ray photoelectron spectroscopy, and chemical bonding is analysed for a series of MXenes, a new family of two-dimensional materials with many applications in nanotechnology.

## Graphical abstract

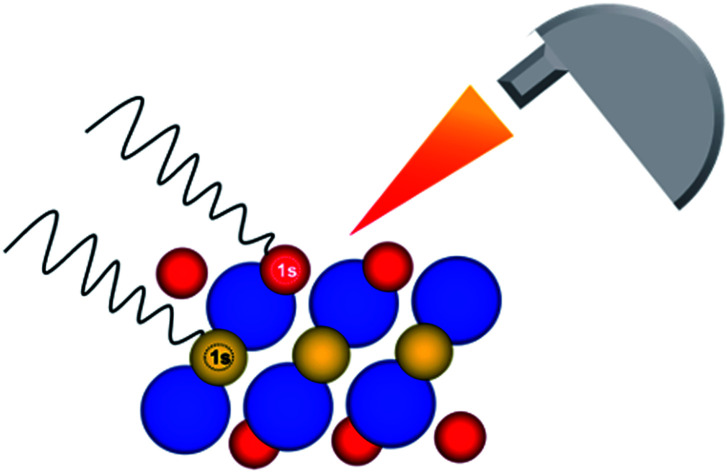

## 1. Introduction

The term MXenes stands for a rapidly growing new family of two-dimensional (2D) materials discovered in 2011 by Naguib *et al.*^1,2^ This new class of materials involves transition metal carbides, nitrides, and carbonitrides which are commonly obtained from chemical exfoliation and sonication of layered ternary carbide or nitride precursors usually referred to as MAX phases.^3–5^ These exhibit a general M_*n*+1_AX_*n*_ chemical formula where *n* indicates the number of atomic layers (*n* = 1, 2 and 3), M corresponds to an early transition metal, A involves an element from groups 13 or 14 such as Al or Si, and X denotes C or N. From a given MAX phase the corresponding MXene with general M_*n*+1_X_*n*_ chemical formula is obtained by a selective chemical etching of the M–A bond. However, because of the synthesis conditions, the MXene surfaces are always functionalized. To take this feature into account it is usual to employ the M_*n*+1_X_*n*_T_*z*_ notation, where T_*z*_ represents a mixture of possible functional groups such as O, F, OH, Cl, and H.^6,7^ Nevertheless, cleaning procedures^8^ and new synthetic methods^9^ are being developed that allows one engineering surface terminations so that, eventually, bare MXenes could be obtained and subsequent applications envisioned.

Because of the unique electric and ionic conduction, optical, plasmonic, and thermoelectric properties exhibited by MXenes,^10^ a rather large number of applications have been envisaged.^6^ Initially, these were mostly related to ion batteries, specifically as supercapacitors,^11^ and energy storage.^2^ However, the number and type of applications have been rapidly growing including, for instance, water purification,^12^ lubrication, gas and energy storage, reinforcement for composites, gas- and biosensors, electromagnetic interference shielding, among other, as summarized in several recent reviews,^13,14^ an exhaustive monography^15^ and other recent studies including a detailed theoretical description of O-covered MXenes.^16^ Furthermore, MXenes have also been proposed as efficient systems in electrocatalysis although with increasing experimental and theoretical studies related to applications in heterogeneous catalysis.^17^ This broad number of application has triggered several theoretical studies, mostly aimed at studying mechanical and electrical properties,^15^ mechanism in catalysis and also to use computational screening to unravel the surface structure of functionalized MXenes^18^ or to propose new MXenes for energy applications.^19^ In spite of all these efforts, fundamental studies have essentially focused on the nature of the chemical bond in MXenes and trends along similar materials are still lacking.

Due to the broad diversity of MXenes, arising from the combination of composition and surface functionalization, it is important to understand the influence of both chemical variables on the resulting chemical bonding and electronic structure. This information is crucial when aiming at using these materials as heterogenous catalyst^17^ or as supports for single atom catalysts (SACs) as recently proposed.^20,21^ There is experimental evidence that, in general, MXenes exhibit a metallic character,^22,23^ as their MAX precursors,^24^ although computational studies show that both bare and functionalized MXenes exhibit strong covalent bonding between the transition metal and the X element and also between the transition metal and the surface chemical groups.^25^ This allows one to modulate the electronic properties of these materials through termination and intercalation.^26^

Regarding the nature of the chemical bond in these materials, most of the available information is extracted from standard density of state plots arising in turn from the band structure provided by periodic density functional theory (DFT) based calculations.^25,27^ However, apart from these general features, there is no systematic information regarding trends in the chemical bond in these materials and, more importantly, on how to identify these trends by suitable experiments. Clearly, a more quantitative, scientifically sound description of the chemical bond in these materials and of trends along the series is needed. X-ray Photoelectron Spectroscopy (XPS) is a widely used analytical technique applicable to different types of materials and exhibits surface sensitivity.^28,29^ Most often, XPS is routinely used for materials characterization since it determines the core level binding energy (CLBE) of elements which can be univocally assigned. The intensity of the XPS peaks allows to determine the concentration of the constituents. Precisely, XPS has been recently used to explore the surface termination of a series of MXenes with several relevant conclusions regarding the composition of these systems and the effect of the number of atomic layers.^30^ This technique has also been used to characterize a WC_1.33_ MXene with ordered carbon vacancies.^31^ Here, it is important to point out that, in addition to chemical analysis, the shift of a given CLBE with respect to a well-defined reference (ΔCLBEs) may be used to assign oxidation states or to provide information regarding the chemical environment of the core ionized atom, and hence extract information about the underlying chemical bond in the material analyzed. This is because, in general, it is possible to use theoretical method to determine the physical origin of the measured ΔCLBEs. Bagus and coworkers have pioneered the study of the relationship between measured ΔCLBEs and properties defining the type of chemical bonding in molecules, surfaces and solids: see reviews in ref. 32–34. They have provided compelling evidence that ΔCLBEs are dominated by charge transfer, polarization, hybridization and electric field effects with charge transfer being often the dominant and the one chosen to analyze in the present work.

While the use of XPS for materials characterization—qualitative and quantitative analysis as well as determining oxidation state—is done in an almost routine way, the use of XPS to extract information from the chemical bond in the material of interest requires some additional, deeper analysis, usually relying on unbiased first-principles theoretical based calculations. The aim of the present work is to present this analysis for a series of bare and oxygen covered MXenes with M_2_C and M_2_CO_2_ general formula, respectively. To this end, we first analyze the trends in the calculated C(1s) in the bare M_2_C MXenes using graphene as a reference and will provide evidence of the relationship between the ΔCLBEs that can be experimentally measured and net charge on the atoms derived from analysis of electron density. Note that this reference is selected on the basis of the analogous atomic arrangement of carbon layer in MXenes. Next, we focus on the trends for the C(1s)–O(1s) CLBEs for the O-terminated M_2_CO_2_ MXenes where a direct comparison between theoretical and experimental values is feasible. Here, a good agreement between theory and experiment is found, provided final state effects are taken into account, which validates the overall computational approach. Finally, we will provide information on how the presence of an oxygen termination influences the charge density distribution and how this can be visualized from XPS measurements.

## 2. Material models and computational details

**Fig. 1:**
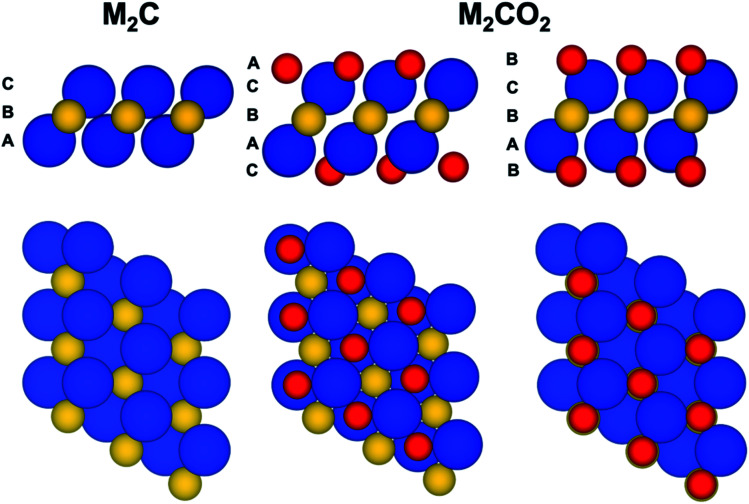
Side and top views of bare M_2_C(0001) and O-functionalized M_2_CO_2_(0001) MXene surfaces. Two different structures of oxygenated MXene are depicted, a fcc-type O termination where every O atom is at the fcc-hollow site with a stacking sequence ACBAC, and a hcp-type O termination where every O atom is at the hcp-hollow site with a stacking sequence BCBAB. Blue, yellow and red spheres correspond to early-transition metal (Ti, V, Cr, Zr, Nb, Mo, Hf, Ta, and W), carbon, and oxygen atoms, respectively.

The present work focuses on a series of bare M_2_C(0001) and O functionalized M_2_CO_2_(0001) MXene surfaces with M = Ti, Zr, Hf, V, Nb, Ta, Cr, Mo, and W (Fig. 1). Each one of these systems is represented by a periodic model with a unit cell replicated in the three dimensions of space. Since the materials are genuinely two-dimensional, a vacuum width of 10 and 16 Å is included for M_2_C and M_2_CO_2_ systems, respectively, to avoid the interaction between the replicas. The smallest sandwich-like unit cell for the M_2_C(0001) systems contains just three atoms—two M and one C—whereas that of the M_2_CO_2_(0001) systems contains five atoms. To perform the structural relaxation, we choose a large 3 × 3 supercell. The purpose of this choice is two-fold. First, to avoid any possible bias introduced by a too strict the periodic symmetry, especially in the O-terminated MXenes, even if this naturally appears in the calculations. Second and more important, to ensure that only one atom (either C or O) is core ionized in the final state calculations (*vide infra*) thus minimizing the interaction with the core holes. These supercells contain 27 and 45 atoms for M_2_C(0001) and M_2_CO_2_(0001) systems, respectively. In spite of using this larger supercell, the structural relaxation using the density functional theory described below converged to a situation where the 1 × 1 periodicity is maintained. Hereafter we will refer to M_2_C(0001) and M_2_CO_2_(0001) surfaces simply as M_2_C and M_2_CO_2_.

Spin-polarized DFT based calculations were carried out using the Perdew–Burke–Ernzerhof (PBE),^35^ a density functional of the Generalized Gradient Approximation (GGA) family, to describe the exchange–correlation potential which is known to accurately describe transition metal carbides,^36^ transition metals bulk,^37,38^ and surfaces.^39^ Since we are concerned with the bulk properties of these materials, dispersion was not included. Note that in the case of O-terminated MXenes, the O atoms are strongly bonded to the MXene surface with adsorption energies larger than 7 eV. All calculations were performed by using the Vienna Ab initio Simulation Package (VASP)^40–42^ where the Kohn–Sham equations are solved in a plane-wave basis set and the interaction between the valence electron density and the atomic cores is described through the Projector-Augmented Wave (PAW) method.^43^ The plane wave expansion was defined by a kinetic energy cut-off of 415 eV, high enough to consider the results converged with the basis set within 1 meV. The numerical integrations in reciprocal space were carried out using a Monkhorst–Pack^44^ grid of 5 × 5 × 1 special *k*-points. The geometry optimizations are considered converged when the forces acting on the nuclei are all below 0.01 eV Å^−1^.

For the different optimized structures, a topological study of the charge density based on the Bader analysis^45^ has been carried out to estimate net atomic charges that will be thereafter used to correlate with the core level binding energy shifts obtained as indicated in the following section. The Bader charges were computed using the code provided by Henkelman *et al.*^46^ which is linked to VASP package.

## 3. Approaching the core level binding energies

For the M_2_C optimized structures, we focused on the C(1s) CLBE and for the M_2_CO_2_ ones, both C(1s) and O(1s) were investigated. Strictly speaking, CLBEs correspond to the total energy difference between the neutral and core ionized systems as in eqn (1)

1 CLBE*_i_* = *E*(*N* − 1, *i*-level) − *E*(*N*)

where *E*(*N*) and *E*(*N* − 1, *i*-level) are the total energy of the neutral system and of that system with one electron removed from the *i*th level. Note that the computed CLBEs in eqn (1) are positive and can be directly compared to XPS measured values. For atoms and molecules, this is a common practice and the procedure is usually referred to as ΔSCF as it implies taking the energy difference of two variational quantities each obtained through a self-consistent field approach.^32–34^ For molecular systems involving first-period elements, the CLBEs thus computed from Hartree–Fock (HF) or hybrid DFT methods are in very good agreement to experiment with PBE performing slightly worse than HF and PBE0 and TPSS performing slightly better.^47^ Nevertheless, as far as shifts are concerned, the effect of the functional is even smaller with absolute errors below 0.5 eV, a relatively small figure taking into account that CLBEs for these elements are in the 200–600 eV range.^48,49^ Importantly, the effect of the density functional on the error in CLBEs with respect to a given reference, the so-called CLBE shifts (ΔCLBEs), is even smaller, a fact that supports using theoretical predictions, with either HF or a suitable DFT method, for XPS interpretation beyond analytical determination.^32–34,47^ In the case of dealing with surfaces and solids, the ΔSCF approach can still be used, provided the extended system is represented by a suitable embedded cluster model. For periodic models, the ΔSCF can only be applied by introducing some approximations such as using sufficiently large supercells to minimize hole–hole interaction, adding a compensating uniform background to provide a neutrally charged unit cell, by promoting one electron from the core to the conduction band instead of the vacuum or by using the transition state approaches that involve half occupation of the core hole.^50^

Even if for molecular systems the agreement between ΔCLBEs predicted by ΔSCF calculations and experiment is generally excellent, it is important to realize that the information regarding ΔCLBEs arises from the core-ionized systems. Thus, one may wonder whether using ΔCLBEs to extract information regarding the neutral systems is justified, an important question that routine use of XPS often disregards. The answer to this question is provided by theory through the decomposition of CLBEs and ΔCLBEs into initial and final state effects,^32–34,51^ the former corresponds to the value of either CLBE or ΔCLBE that will be obtained using for the ionized system strictly the wavefunction (or the electron density) of the neutral one with just one electron less. The importance of the decomposition of CLBEs and ΔCLBEs into initial and final state contributions has been highlighted recently in the framework of the so-called equivalent core model.^51^

The difference between initial and final state CLBEs is the relaxation energy, the gain in energy due to the response of the electron density to the presence of the core hole. The relaxation energy may be large, of the order of 10–30 eV for the 1s core hole in first period atoms. However, there is compelling evidence that, to a large extent, the relaxation energy is a quite local atomic quantity. As a result, for a broad number of materials, ΔCLBEs are dominated by initial state effects. This statement is important because, on the one hand, justifies the use of XPS to analyze the chemical bond of the materials in the neutral unionized state and, on the other hand, provides a rather simple way to analyze ΔCLBEs in solids.

In the case of using the HF method, Koopman's theorem^52^ ensures that the energy difference leading to the initial state contribution to the CLBE is equal to minus the orbital energy from which the electron is removed. This provides a very useful framework for the interpretation of CLBEs in terms of orbitals as recently highlighted.^53^ In the case of using DFT, the Kohn–Sham orbital energies do not provide an approximation to the absolute CLBE value, but it has been shown that these orbital energies provide reliable trends for ΔCLBEs and, hence, can be used to provide initial state estimates.^54^

In the present work, we will rely on both the initial state and final estimate of CLBEs and ΔCLBEs for C(1s) in M_2_C, and C(1s) and O(1s) for M_2_CO_2_. Unless specified, we will use ΔCLBEs to refer to initial state values. The initial state CLBEs are estimated from the Kohn–Sham orbital energies corresponding to the PAW as implemented in VASP and referred to the Fermi level of each material as in eqn 2

2 CLBE_*i*_ = −[*ε*_*i*_ − *ε*_F_],

where *ε*_*i*_ and *ε*_F_ are the Kohn–Sham orbital energy for the corresponding core level “*i*” and the Fermi energy, respectively. This is a necessary reference for a proper comparison between different systems, but it is not necessary when comparing two core levels in a given system such as C(1s) and O(1s) in M_2_CO_2_ MXene.

Final state effects are included using one of the different formalisms also implemented in VASP. This relies on generating a core excited ionic PAW on the fly.^55^ A survey of the reliability of this and closely related methods in predicting ΔCLBEs of a large database of gas phase molecules has been recently reported.^56^ In that work, it has been shown that the so called Janak–Slater (JS^*n*^) transition state method,^57,58^ accurately predicts ΔCLBEs in the mentioned dataset of molecules. The JS^*n*^ approach considers a final state where the core and vacuum levels have a half occupation. Here we use the same JS^*n*^ formalism and, to find additional technical details, the interested reader is addressed to ref. 56.

## 4. Results for M_2_C bare MXenes

The calculated values for the C(1s) CLBEs of the set of bare MXenes considered in the present work, relative to the Fermi level of each system, are compiled in Table 1, where *ε*_*i*_ and *ε*_F_ values are reported for completeness. These are initial and final state values as they are derived from the core Kohn–Sham orbital energies (*ε*_*i*_ in eqn (2)) as represented by the PAW method. Table 1 also reports the calculated Bader charge on the C atom of each MXene. Inspection of the results in Table 1 indicates that there are several features that need to be discussed. First, all C(1s) CLBE values are grouped in the 265–266 eV range, these values are excessively low as reported experimental values for MXenes are typically around 282 eV although this is somehow arbitrary since relies on having the analyzer calibrated to that the C(1s) of free C adjusted to 284.6 eV.^59^ Nevertheless, the range for C(1s) in bulk carbides with respect to a different experimental reference; namely the valence band edge which provides a more sound and reproducible reference, is also around 282 eV.^60,61^

**Table 1:** Kohn–Sham orbital energy (*ε*_1s_), Fermi energy (*ε*_F_), initial and final state C(1s) CLBE (IS-CLBE and FS-CLBE respectively) relative to the Fermi energy of each system (see eqn (2)) and net charge on the C atom (*Q*_C_) of bare M_2_C MXenes. The number of d electrons in the corresponding metal (d^*n*^) is also provided. The units of energetic parameters (*ε*_1s_, *ε*_F_, and CLBE) are in eV, whereas *Q*_C_ units are |e|

MXene	d^*n*^	*ε*_1s_ (IS)	*ε*_1s_ (FS)	*ε*_F_	IS-CLBE	FS-CLBE	*Q*_C_
Ti_2_C	d^2^	−265.4	−290.6	−0.3	265.1	290.3	−2.3
Zr_2_C		−265.9	−291.0	−0.7	265.2	290.3	−2.2
Hf_2_C		−265.7	−290.7	−0.7	265.0	290.0	−2.5
V_2_C	d^3^	−265.8	−291.4	−0.3	265.5	291.1	−2.0
Nb_2_C		−266.8	−291.9	−0.7	266.1	291.2	−1.9
Ta_2_C		−267.0	−292.6	−1.2	265.8	291.4	−2.2
Cr_2_C	d^4^	−267.4	−292.8	−1.6	265.8	291.2	−1.7
Mo_2_C		−267.6	−292.3	−0.9	266.7	291.6	−1.6
W_2_C		−267.4	−292.7	−1.0	266.4	291.7	−1.4

The underestimation of the initial state calculated C(1s) CLBEs of MXenes based on the Kohn–Sham orbital energies has two origins. One is general for all computational approaches and comes from the fact that, differently from the case of molecular systems where experimental and calculated CLBEs are referred to the vacuum level, the calculated values for periodic systems are referred to the calculated Fermi energy of the material, this is precisely the reason why values in Table 1 are referred to the Fermi energy of each MXenes (*cf.*eqn (2)). The experimental reference may also vary and may rely on Fermi level as measured from photoemission as the top of the valence band, as in the work of Rodriguez *et al.*^60^ for transition metal carbides and by Halim *et al.*^30,62^ for some MXenes. Yet, for convenience, some previous work calibrated the analysis with respect to some well-known references such as the so-called adventitious carbon^63^ usually adjusted at 284.6 eV; this is the case of the experiments reported by Naguib *et al.* for the Nb_2_C MXene.^59^ It is worth noting that, in the case of using the photoemission measured Fermi level, the adventitious carbon is found around 283–284 eV.^64^ Hence, for analytical purposes, both approaches lead to similar conclusions. The second cause as the different reference simply comes from the fact that the Koopman's theorem does not apply to DFT calculations and, hence, Kohn–Sham orbital energies do not provide an estimate of the initial state CLBEs.^65^ Better estimates can be obtained when including final state effects^32,33,50^ although, because of the reference problem mentioned above, a comparison to experiment based on absolute CLBEs values remains difficult. Nevertheless, one must point out that, in some cases, accounting for final state effects is mandatory as we will show in the following section.

To avoid problems originated from the choice of a given reference, the common choice is to rely on ΔCLBEs, *i.e.* shifts of the core level of interest. In addition, this allows using the Kohn–Sham orbital energies since, even if these do not represent the CLBEs, they provide a good estimate of ΔCLBEs.^65^ In the present case we use graphene as a reference since, in addition, provides a system where C atoms have all a zero net charge. The graphene initial state for the C(1s) CLBE estimated from the Kohn–Sham orbital energies and relative to the Fermi energy is 265.7 eV, again much too low compared to the experimental value for graphene at 284.6 eV. The hypothesis here is that the error in graphene and MXenes is similar so that the calculated initial state ΔCLBEs can be taken as rather accurate. Below we will present results including final state effects that provide additional arguments supporting this statement.

**Fig. 2:**
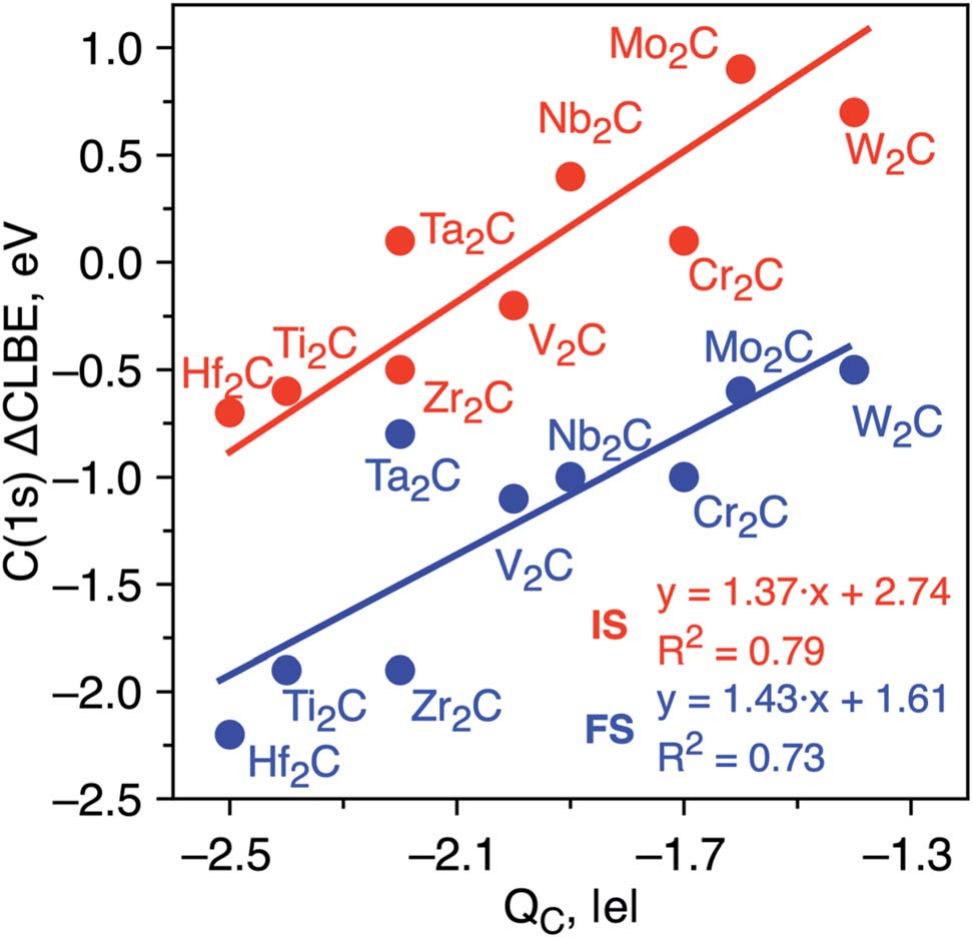
Initial state (red dots) and JS^*n*^ final state (blue dots) core level binding energy shifts (ΔCLBE in eV) for the C(1s) in bare M_2_C MXenes *versus* the Bader charge in the C atom (*Q*_C_ in |e|). All values are relative to the equivalent C(1s) CLBE in graphene (265.7 eV and 292.2, respectively) and have been estimated from eqn (2). Solid red and blue lines correspond to each linear fitting. The regression equations for the initial state and JS^*n*^ approaches are ΔCLBE = 1.37*Q*_C_ + 2.74 (*R*^2^ = 0.8) and ΔCLBE = 1.43*Q*_C_ + 1.61 (*R*^2^ = 0.7), respectively.

The differences in the C(1s) initial state CLBEs for each one of the MXenes in Table 1 are small, thus indicating that the ΔCLBEs are also small. In fact, all initial state ΔCLBEs are in the [−0.7, +0.7] eV interval. Here, one may wonder whether these differences are significant and whether one can provide a sound scientific explanation for the predicted variance. Several factors are known to contribute to the ΔCLBEs of a given core level of a given element which include charge transfer, hybridization and coordination, among others with the first one providing by far the largest contribution, to the point the rest are often, sometimes incorrectly, neglected.^32–34^ To investigate whether charge transfer is at the origin of such differences in the calculated initial state ΔCLBEs, we present the correlation between C(1s) initial and final state ΔCLBEs and the Bader charge in C atom as shown in Fig. 2. Indeed, a rather good linear trend with a *R*^2^ parameter close to 0.8 is found confirming such correlation for initial and final state, respectively. It is worth pointing out that taking the initial state ΔCLBEs directly from the Kohn–Sham values without taking into account the Fermi energy also leads to a linear trend although with *R*^2^ = 0.7. Taking the values relative to the Fermi level provides not only a more realistic comparison to experiment but also a better relationship with respect to the charge in the core ionized atom.

To determine whether the inclusion of final state effects affects this correlation, the ΔCLBE values have been calculated using the JS^*n*^ approach above discussed with results shown in Fig. 2. The linear trend is preserved with *R*^2^ = 0.7 which confirms that differences in the net charge in the MXene atom originate observable shifts in the C(1s) CLBE. This is a relevant conclusion as it shows that a fine analysis of the XPS features in MXenes provides valuable information about trends in the chemical bond in these novel materials. It is worth noting that the trends evidence three groups that correspond precisely with the series along the periodic table. However, even for a given triad such as Ti, Zr and Hf, there are slight differences in the ΔCLBE and in the net charge in C that deserve a more accurate and detailed analysis. In the next section, we investigate whether similar conclusions hold for the oxygen covered MXenes.

## 5. Results for oxygen covered M_2_CO_2_ MXenes

To investigate the optimum structure of the oxygen terminated MXenes, two different structures of oxygenated MXene have been considered. In the first one, hereafter referred to as fcc-type, the O atoms locate above the three-fold hollow sites formed by the M atoms (a fcc-hollow site) whereas in the second one (hcp-type) the O atoms are located in the hollow-site directly above a C atom in the middle layer (a hcp-hollow site) (Fig. 1). After full structural relaxation, the total energies revealed that fcc-type structure is preferred for the MXenes composed by Ti, Zr, Hf, V, Nb and Ta transition-metal atoms whereas a hcp-type structure is more favorable those MXenes composed by Mo, W, with the case of Cr being somehow in between but closer to a fcc-type structure, in agreement with previous findings.^66–71^

Before attempting to find correlations similar to that described in the previous section, we focus on a comparison between calculated and experimental values which is well possible for these systems because one can just focus on the difference between the C(1s) and O(1s) CLBEs which does not require any additional reference. From the experimental side, this shift corresponds simply to the difference between the two experimental peaks whereas from the computational modelling side one can just rely on the initial state CLBE values estimated from the Kohn–Sham orbital energies without needing to refer them to the Fermi energy which is common for the two core levels. Unfortunately, experimental results are only available for Ti_2_CO_2_, V_2_CO_2_, Nb_2_CO_2_, Cr_2_CO_2_ and MoCO_2_ and, in some cases, the termination is not pure O but also involves OH groups (see Table S1 in ESI† for further details).^72–75^ These structural differences induce changes on the resulting XPS analysis. Nevertheless, these cases suffice to check the reliability of the present computational approach. Table 2 compiles the available experimental core level binding energy shifts between the C(1s) and O(1s) peaks in these five MXenes *versus* the equivalent calculated initial (IS) and JS^*n*^ final states (FS) estimations. Experimental values are taken from the literature summarized in Table S1† where the reported tentative assignment is also included; further details about experiments can be found in ref. 72–76. Let us start by analyzing the initial state predictions where C(1)–O(1s) shifts are systematically underestimated with respect to experimental values regardless of the MXene composition. The average difference between IS and experimental values is of ∼8 eV with Cr_2_CO_2_ and Nb_2_CO_2_ being he lower and upper cases with differences of 5.9 and 9.1 eV, respectively. These differences have a clear physical explanation which is directly related to the fact that the calculated values do not account for the relaxation energy in response to the presence of the core hole whereas the experimental values do obviously include these effects. To further confirm that this is the case, calculations have been carried out including the final state effects for both cores following the JS^*n*^ approach. Our previous statement is confirmed by analyzing the energetic difference Δ(FS − IS) listed in Table 2 of ∼9 eV. Clearly, the inclusion of final state effects values gives much closer to the experimental one as shown in the Δ(FS − experiment) column in Table 2. For instance, for Ti_2_CO_2_ the IS difference between C(1s) and O(1s) CLBEs of 241.7 eV, becomes 250.5 eV once final state effects are included. This is in good agreement with experimental shift of 249.4 eV; the difference being due to the approximate nature of the JS^*n*^ approach. Yet, in relative terms, the error with respect to experiment is reasonably small with the exception of Cr_2_CO_2_ where further analysis may be necessary. Besides, inclusion of final state effects confirms that the difference in the initial state shift with respect to experiment arises from the different relaxation of the C and O atoms induced by the presence of the core hole, a largely atomic effect.^32,33^ Upon inclusion of approximate final state effects, the majority of calculated results are now much closer to experiments, which further support the conclusion of the present work. Yet, we must point out that the C(1s)–O(1s) shift for Ti_2_CO_2_ and V_2_CO_2_ is significant while available experimental values are closer. Including final state effects by means of the JS^*n*^ approach does not improve the agreement with experiment. The fact that the agreement is quite good for the other cases suggests that the cases of V_2_CO_2_ and Cr_2_CO_2_ need to be revised in detail by both experiments, with regard to possible problems in the sample such as surface coverage effects, and theory, with a more accurate treatment of final state effects and considering coverage effects as well.

**Table 2:** Calculated initial state (IS), final state (FS) and available experimental values (exp) for the C(1s)–O(1s) CLBEs in M_2_CO_2_ (M = Ti, V, Cr, Nb, and Mo). Δ(FS − IS) stands for difference between IS and FS values, which is almost constant, whereas Δ(FS − Exp) correspond to the difference between FS and experimental values from the references Exp column. Further details are given in Table S1. Numbers in brackets correspond to references. All values are in eV

MXene	IS	FS	Exp	Δ(FS − IS)	Δ(FS − Exp)
Ti_2_CO_2_	−241.7	−250.5	−249.4 (ref. 73)	8.8	−1.1
			−249.2 (ref. 76)		−1.3
V_2_CO_2_	−241.0	−250.9	−249.5 (ref. 72)	9.9	1.4
			−249.2 (ref. 76)		1.7
Cr_2_CO_2_	−241.0	−249.6	−246.9 (ref. 75)	8.6	2.7
Nb_2_CO_2_	−240.9	−249.4	−250.0 (ref. 72)	8.5	0.6
			−248.8 (ref. 76)		−0.6
Mo_2_CO_2_	−240.5	−248.9	−248.0 (ref. 74)	8.4	0.9
			−248.4 (ref. 76)		0.5

The preceding discussion reinforces the hypothesis that core level shifts are dominated by initial state effects also in MXenes. This provides a useful tool to investigate the chemical bond in these systems and support the trend observed for bare MXenes relating ΔCLBEs values for the C(1s) level and net charge on the C atom. To investigate whether this trend holds for the O-terminated MXenes, Tables 3 and 4 report the initial state and final state ΔCLBEs values for the C(1s) and O(1s) levels, all referred to the Fermi level of each system, relative to the C(1s) in graphene, also referred to the graphene Fermi level, and the net charge on the C and O atom, respectively. Unexpectedly, the analysis of the values on these tables does not show any clear correlation between other than a rough trend suggesting that large ΔCLBEs values are accompanied by large values of the net charge on the core ionized atom. The lack of a clearer correlation indicates that, in the O-terminated MXenes, the charge is not the dominant factor governing the ΔCLBEs and, consequently, that ΔCLBEs do not provide quantitative information about the charge state of the atoms in these materials. A possible explanation for the lack of correlation with the atomic net charge is that variations in interatomic distances also contribute to the ΔCLBEs as recently noted.^51^ In fact, O-termination induces a significant relaxation of the MXene atomic structure.

**Table 3:** Kohn–Sham orbital energy (*ε*_1s_), Fermi energy (*ε*_F_), initial and final state C(1s) CLBE (IS-CLBE and FS-CLBE respectively) relative to the Fermi energy of each system (see eqn (2)) and net charge on the C atom (*Q*_C_) of full relaxed O-terminated M_2_CO_2_ MXenes. The number of d electrons in the corresponding metal (d^*n*^) is also provided. The units of energetic parameters (*ε*_1s_, *ε*_F_, and C(1s) CLBE) are in eV, whereas *Q*_C_ units are |e|

MXene	d^*n*^	*ε*_1s_(IS)	*ε*_1s_(FS)	*ε*_F_	IS-CLBE	FS-CLBE	*Q*_C_
Ti_2_CO_2_	d^2^	−265.7	−290.7	−1.0	264.7	289.7	−1.7
Zr_2_CO_2_		−265.2	−290.3	−1.1	264.1	289.2	−1.9
Hf_2_CO_2_		−264.7	−289.5	−0.9	263.8	288.6	−2.1
V_2_CO_2_	d^3^	−267.0	−292.2	−1.5	265.5	290.7	−1.6
Nb_2_CO_2_		−266.8	−291.9	−0.9	265.9	291.0	−1.8
Ta_2_CO_2_		−266.2	−291.6	−0.3	265.9	291.3	−2.1
Cr_2_CO_2_	d^4^	−267.1	−292.1	−1.5	265.6	290.6	−1.3
Mo_2_CO_2_		−267.3	−292.3	−1.2	266.1	291.1	−1.3
W_2_CO_2_		−266.3	−291.7	−0.8	265.5	290.9	−1.5

**Table 4:** Kohn–Sham orbital energy (*ε*_1s_), Fermi energy (*ε*_F_), initial state and final state O(1s) CLBE (IS-CLBE and FS-CLBE respectively) relative to the Fermi energy of each system (see eqn (2)) and net charge on the O atom (*Q*_O_) of O-terminated M_2_CO_2_ MXenes. The number of d electrons in the corresponding metal (d^*n*^) is also provided. The units of energetic parameters (*ε*_1s_, *ε*_F_, and O(1s) CLBE) are in eV, whereas *Q*_O_ units are |e|

MXene	d^*n*^	*ε*_1s_(IS)	*ε*_1s_(FS)	*ε*_F_	IS-CLBE	FS-CLBE	*Q*_O_
Ti_2_CO_2_	d^2^	−507.4	−541.3	−1.0	506.4	540.3	−1.2
Zr_2_CO_2_		−507.1	−541.2	−1.1	506.0	540.1	−1.2
Hf_2_CO_2_		−507.0	−538.6	−0.9	506.1	537.7	−1.3
V_2_CO_2_	d^3^	−508.0	−541.5	−1.5	506.5	540.0	−1.0
Nb_2_CO_2_		−507.6	−541.3	−0.9	506.7	540.4	−1.1
Ta_2_CO_2_		−507.4	−541.3	−0.3	507.1	541.0	−1.2
Cr_2_CO_2_	d^4^	−508.1	−541.6	−1.5	506.6	540.1	−0.9
Mo_2_CO_2_		−507.8	−541.2	−1.2	506.6	540.0	−1.0
W_2_CO_2_		−507.5	−541.4	−0.8	506.7	540.6	−1.0

**Fig. 3:**
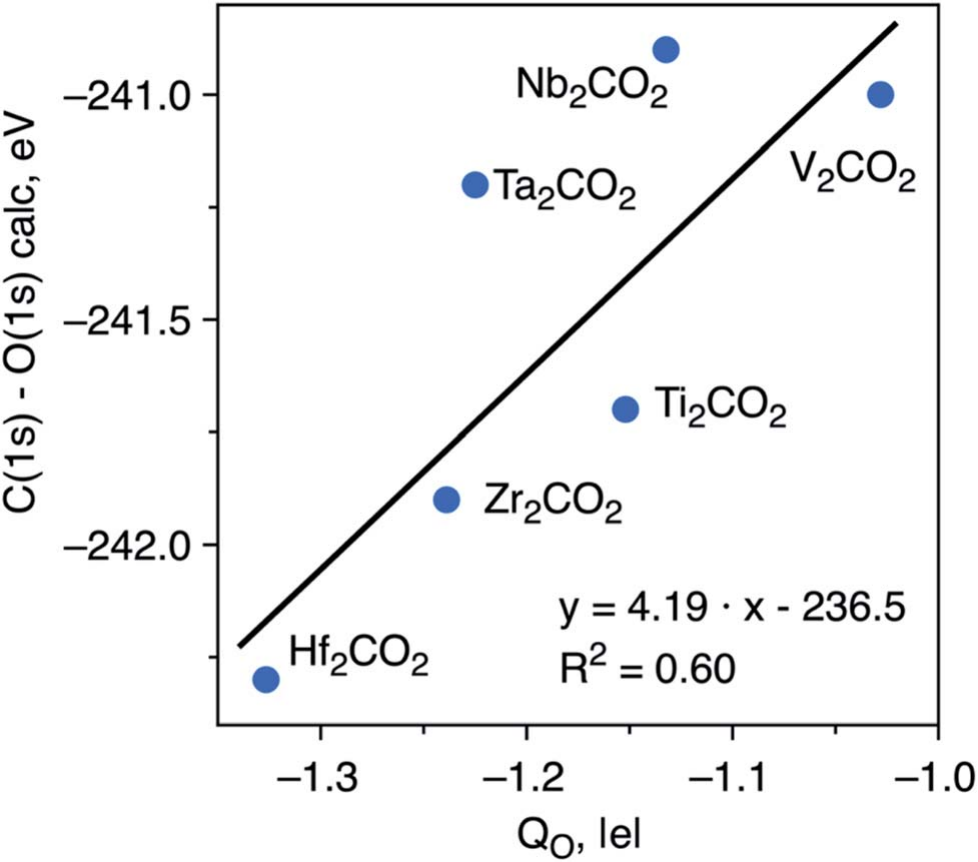
Correlation between initial state ΔCLBEs C(1s) and O(1s) of relaxed fcc-type O functionalized M_2_CO_2_ (M = Hf, Zr, Ti, Ta, V, and Nb) structures with the Bader charge in the O atom (*Q*_O_). Solid black line corresponds to the linear fitting.

To try to shed some light on the trends in ΔCLBEs for the C(1s) in the M_2_CO_2_ MXenes, we analyze the correlation between ΔCLBEs for the C(1s) and the net charge in C (*Q*_C_) along the groups and along the periods of the periodic table. For the M_2_CO_2_ where M = Ti, Zr, Hf, the correlation between ΔCLBEs for C(1s) and *Q*_C_ is excellent with *R*^2^ = 0.93 although it gets slightly worse for M = V, Nb, Ta with *R*^2^ = 0.81 but with a change in the slope from positive to negative, which is not so clear to understand because in one case larger ΔCLBEs imply larger *Q*_C_ whereas in the other case larger ΔCLBEs imply smaller *Q*_C_. Finally, the trend between ΔCLBEs and *Q*_C_ becomes totally meaningless for M = Cr, Mo, W; the latter is not surprising if one realizes that in the last triad there is a change in the adsorption mode of O with respect to the two other triads and also that Cr occupies a site which is different in the case of Mo and W. Similarly, a clear trend is found between these two magnitudes along the period although with significantly smaller *R*^2^ values which get considerably worse when going from the 3d to the 5d metals, a behavior which is also attributed to the change of adsorption site for atomic O. Similar trends are found when comparing ΔCLBEs for O(1s) and *Q*_O_. Finally, plotting the C(1s) and O(1s) initial state ΔCLBEs with respect to the net charge of the O atom in the relaxed M_2_CO_2_ with fcc-type structure (Fig. 3) results in a poorer correlation than that depicted in Fig. 2. This indicates that C(1s) and O(1s) ΔCLBEs in the M_2_CO_2_ structure are not governed solely by the charge but include structural effects as well which has implications for the interpretation of XPS data.

## 6. Conclusions

A computational study based on periodic density functional theory calculations reveals that the trends in the C(1s) core level binding energy shifts in bare MXenes of M_2_C stoichiometry with M = Ti, Zr, Hf, V, Nb, Ta, Cr, Mo, and W are dominated by initial state effects and are directly related to the net charge on the C atom. Consequently, XPS measurements can be used to extract information regarding the oxidations state of these materials.

The good agreement between experimental and calculated shifts between C(1s) and O(1s) core level binding energies, especially when final state effects are included to account for the difference in relaxation energy induced by the core holes, provides further support to the use of initial state values to investigate the trends in these quantities.

For the O-terminated MXenes, the correlation between core level shift, either C(1s) or O(1s), and the net charge in the core ionized atom, either C or O, is much less significant than for the bare MXenes which is attributed to structural changes induced by the presence of O and to the fact that the O adsorption site may vary. This is confirmed by the excellent correlation between the C(1s) and O(1s) ΔCLBEs and the net charge of the O atom along the Ti, Zr, Hf and V, Nb, Ta where the fcc-type O functionalization dominates.

The fact that the correlations between C(1s) core level shifts and net charges in C of the O-terminated MXenes are dependent on the period or group of the periodic table, largely because of changes in the oxygen adsorption site and concomitant structural changes, implies that conclusions about the chemical bond extracted from XPS measurements need to the taken with extreme care. Here, information provided from theoretical approaches is required to extract physically meaningful conclusions.

The present conclusions have been extracted for M_2_C MXenes but, in the view of the unraveled physical mechanisms and trends, it is anticipated that they will also hold for other MXenes although the increase in the number of atomic layers may result in some additional effects that need to be investigated.

## Author contributions

F. I. planned the work, F. I. and A. M. G. wrote the manuscript. N. G. R and M. K. prepared the models and carried out the calculations. A. M. G. and N. G. R. prepared the figures. All authors contributed to the analysis of results, scientific discussion, elaborated the conclusions and have revised the final version of the manuscript.

## Conflicts of interest

There are no conflicts to declare.

## Supplementary Material

NA-003-D0NA01033B-s001
