# Supplementary material for: Relating X-ray photoelectron spectroscopy data to chemical bonding in MXenes
Source: Nanoscale Adv. 2021 Mar 1;3(10):2793–801. doi: 10.1039/d0na01033b (PMC9418319; doi:10.1039/d0na01033b)
Supplement: NA-003-D0NA01033B-s001 [file NA-003-D0NA01033B-s001.pdf]

Electronic Supplementary Material (ESI) for Nanoscale Advances.
This journal is © The Royal Society of Chemistry 2021

Electronic Supplementary Information (ESI)

Relating X-Ray Photoelectron Spectroscopy Data to Chemical Bonding in MXenes

Néstor García-Romeral,[a] Masoomeh Keyhanian,[a,b] Ángel Morales-García,[a] Francesc Illas[a]*

[a]Departament de Ciència de Materials i Química Física & Institut de Química Teòrica i Computacional (IQTCUB), Universitat de Barcelona, c/Martí i Franquès 1-11,
08028 Barcelona, Spain

[b]Department of Physical Chemistry, Faculty of Chemistry, University of Mazandaran, Babolsar 47416-95447, Iran

e-mail: francesc.illas@ub.edu

Table S1. Experimental results of XPS analysis in MXenes. Bold numbers are selected to compare and discuss our theoretical predictions.

MXene	Region	Binding energy (eV)	Asignation	Ref.
Nb_2CT_z	O (1s)	530.0<br>**531.8**<br>532.4	O in Nb_2O_5<br>**O/HO-Nb_2C**<br>Water trapped between MXene layer	[1]
	C (1s)	**281.8**<br>282.0<br>283.6	**C in Nb_2C**<br>Non-stoichiometric C in Nb_2C<br>Adventitious contamination	
V_2CT_z	O (1s)	529.9<br>**531.3**<br>532.9	Mixed VO_x and surface oxide groups<br>**Mixed VO_x and surface oxide groups**<br>Water trapped between MXene layer	[1]
	C (1s)	**281.8**<br>286.1<br>284.5<br>288.3	**C in V_2C**<br>Adventitious contamination<br>Adventitious contamination<br>Adsorbed methanol	
Ti_2CT_z before sputtering	O (1s)	**531.1**<br>532.0<br>533.1	**C-Ti-O_z**<br>C-Ti-$(OH)_z$<br>Water trapped between MXene layer	[2]
	C (1s)	**281.9**<br>286.2<br>289.0	**C-Ti-T_z**<br>C-O<br>C-OO	
Ti_2CT_z after sputtering	O (1s)	**531.3**<br>532.2<br>533.5	**C-Ti-O_z**<br>C-Ti-$(OH)_z$<br>Water trapped between MXene layer	
	C (1s)	**281.9**<br>286.2	**C-Ti-T_z**<br>C-O	
Mo_2CT_z	O (1s)	**531.1**<br>532.2<br>533.4	**Mo_2CO_x (-O terminated)**<br>$Mo_2C(OH)_x$ (-OH terminated)<br>Water trapped between MXene layer	[3]
	C (1s)	**283.1**	**Mo-C species**	
MultiLayered Cr_2CT_z	O (1s)	530.2<br>531.2	**O-Cr-C (-O terminated)**<br>HO-Cr-C	[4]
	C (1s)	283.2	**C-Cr-T_z**	
FewLayered Cr_2CT_z	O (1s)	**530.4**<br>531.6	**O-Cr-C (-O terminated)**<br>HO-Cr-C	[4]
	C (1s)	**283.5**	**C-Cr-T_z**	

Table S2. Kohn-Sham orbital energy (ε_{1s}), Fermi energy (ε_F), initial state C(1s) CLBE relative to the Fermi energy of each system (see Eq. 2) and net charge on the C atom (Q_C) of partial relaxed O-terminated M_2CO_2 MXenes. The number of d electrons in the corresponding metal (d^n) is also provided. The units of energetic parameters (ε_{1s}, ε_F, and C(1s) CLBE) are in eV, whereas Q_C units are |e|. Note that the O layers are relaxed maintaining the M_2C fixed at the clean MXene.

**MXene**	**d^n**	**ε_{1s}**	**ε_F**	**CLBE**	**Q_C**
Ti_2CO_2	d^2	-267.8	-3.6	264.2	-1.9
Zr_2CO_2		-265.6	-2.0	263.6	-1.9
Hf_2CO_2		-265.2	-1.5	263.7	-1.9
V_2CO_2	d^3	-268.0	-2.7	265.3	-1.6
Nb_2CO_2		-267.6	-2.0	265.6	-1.8
Ta_2CO_2		-267.4	-1.6	265.8	-2.1
Cr_2CO_2	d^4	-267.7	-2.2	265.5	-1.3
Mo_2CO_2		-268.3	-1.7	266.6	-1.4
W_2CO_2		-267.3	-1.5	265.8	-1.5

Table S3. Kohn-Sham orbital energy (ε_{1s}), Fermi energy (ε_F), initial state O(1s) CLBE relative to the Fermi energy of each system (see Eq. 2) and net charge on the O atom (Q_O) of partial relaxed O-terminated M_2CO_2 MXenes. The number of d electrons in the corresponding metal (d^n) is also provided. The units of energetic parameters (ε_{1s}, ε_F, and O(1s) CLBE) are in eV, whereas Q_O units are |e|. Note that the O layers are relaxed maintaining the M_2C fixed at the clean MXene.

**MXene**	**d^n**	ε_{1s}	ε_F	**CLBE**	**Qo**
Ti_2CO_2	d^2	-508.8	-3.6	505.2	-1.0
Zr_2CO_2		-507.4	-2.0	505.4	-1.2
Hf_2CO_2		-507.0	-1.5	505.5	-1.3
V_2CO_2	d^3	-508.8	-2.7	506.1	-1.0
Nb_2CO_2		-508.3	-2.0	506.3	-1.1
Ta_2CO_2		-508.2	-1.6	506.6	-1.2
Cr_2CO_2	d^4	-508.7	-2.2	506.5	-0.9
Mo_2CO_2		-508.6	-1.7	506.9	-0.9
W_2CO_2		-508.2	-1.5	506.7	-1.0

REFERENCES

1 M. Naguib, J. Halim, J. Lu, K. M. Cook, L. Hutman, Y. Gopgotsi, and M. W. Barsoum, *J. Am. Chem. Soc.* **2013**, *135*, 15966-15969.

2 J. Halim, K. M. Cook, M: Naguib, P. Eklund, Y. Gogotsi, J. Rosen, and M. W. Barsoum, *Appl. Surf. Sci.* **2016**, *362*, 406-417.

3 J. Halim, S. Kota, M. R. Lukatskaya, M. Naguib, M.-Q. Zhao, E. J. Moon, J: Pitock, J. Nanda, S. J. May, Y. Gogotsi, and M. W. Barsoum, *Adv. Funct. Mater.* **2016**, *26*, 3118-3127.

4 B. Soundiraraju, R. Raghavan, and B. K. George, *ACS Appl. Nano Mater.* **2020**, *3*, 11007-11016.
